# Effect of Type and Concentration of Ballasting Particles on Sinking Rate of Marine Snow Produced by the Appendicularian *Oikopleura* *dioica*

**DOI:** 10.1371/journal.pone.0075676

**Published:** 2013-09-25

**Authors:** Fabien Lombard, Lionel Guidi, Thomas Kiørboe

**Affiliations:** 1 Centre for Ocean Life, National Institute for Aquatic Resources, Section for Oceanography and Climate, Technical University of Denmark, Charlottenlund, Denmark; 2 UPMC, Université Paris06, UMR 7093, Laboratoire d’Océanographie de Villefranche-sur-Mer, Observatoire Océanologique de Villefranche sur Mer, France; Technical University of Denmark, Denmark

## Abstract

Ballast material (organic, opal, calcite, lithogenic) is suggested to affect sinking speed of aggregates in the ocean. Here, we tested this hypothesis by incubating appendicularians in suspensions of different algae or Saharan dust, and observing the sinking speed of the marine snow formed by their discarded houses. We show that calcite increases the sinking speeds of aggregates by ~100% and lithogenic material by ~150% while opal only has a minor effect. Furthermore the effect of ballast particle concentration was causing a 33 m d^-1^ increase in sinking speed for a 5×10^5^ µm^3^ ml^-1^ increase in particle concentration, near independent on ballast type. We finally compare our observations to the literature and stress the need to generate aggregates similar to those in nature in order to get realistic estimates of the impact of ballast particles on sinking speeds.

## Introduction

Sinking marine particles and especially marine snow are responsible for a significant fraction of the sinking carbon in the ocean [1,2] and thus play an important role in the biological carbon pump [3]. Important efforts have been made to understand how sinking aggregates are formed in surface layers, sink through the water column and are consumed during their path to the bottom (see reviews in [4,5,6]). In particular, sinking speed variations have been attributed to both changes in particle size [7-10], composition, and density [11-13]. The quantity of organic carbon transported to depth by sinking particles has been observed to be closely associated to the quantity of inorganic constituents of the particles [11,12,14]. This inorganic part is mostly composed of opal, calcite, and lithogenic materials originating from, respectively, diatoms, coccolithophores or foraminifers, and atmospheric or river inputs. These constituents play a ballasting role on the particles and increase their sinking velocity because of their high density [15-19]. However, in most studies, marine snow particles were obtained artificially under laboratory conditions using long incubations of dense algal culture in roller tanks, producing particles with characteristics different from those formed *in situ* [20]. Therefore, there is a strong need to confirm these observations with realistic aggregates.

Appendicularians are planktonic tunicates that use external mucous devices, called houses, to filter, concentrate, and feed on particles ranging in size from 0.2 µm to several hundreds of microns [21,22]. These houses can clog, thus forcing the appendicularian to discard the house and secrete a new one several times per day (up to 27 d^-1^ [23]). The discarded houses can include concentrated non-ingested particles such as bacteria, phytoplankton, and lithogenic dusts [17,24,25], while scavenging of additional particles by sinking houses has been shown to only play a minor role in their final composition [26]. These mucous feeding structures are an important source of marine snow formed by zooplankton [27], and may contribute significantly to POC flux [28,29]. Despite this potentially high contribution to carbon flux, only few studies have focused on appendicularians’ impact on POC fluxes [30], and nothing is known about the impact of food or ballasting particles (eg. [31]) on the sinking properties of discarded houses. Appendicularians are easy to cultivate [32,33], and because house production is a biologically controlled process, it allows the production of discarded houses in the laboratory with physical properties similar to those produced in the ocean. Moreover, appendicularian-produced marine snow has sinking characteristics comparable with phytoplankton aggregates [7,27,34], which makes these biological aggregates a good proxy for marine snow particles and suitable for testing the effect of ballast particle composition.

In this study we used the appendicularian *Oikopleura* *dioica* to produce houses loaded with different kind of ballasting material and tested the influence of different concentrations and types of ballasting material on the sinking speed.

## Materials and Methods

Four types of marine snow particles were formed from appendicularian houses that had integrated four types of “ballasting” material. The ballasting particles were obtained through three species of phytoplankton and Saharan dust. The organic ballasting materials were *Isochrysis* *galbana* (flagellate algae), opal ballast was provided by *Thalassosira* *pseudonana* (silicifying diatom), and calcite ballast by *Emiliana* *huxleyi* (calcifying coccolithophorids). All phytoplankton were cultivated in F/2 medium (Guillard) with addition of selenium for *E* *. huxleyi* and under 12h: 12h light cycle (250 *µ*E m^‑2^ s^-1^). The dust particles were collected and treated as described in [35]. The resulting marine snow particles will hereafter be referred to as organic, opal, calcite, and lithogenic particles.

Appendicularians ( *Oikopleura* *dioica* ) were collected in the Øresund strait, western Baltic Sea, and grown in the laboratory following standard protocols [34,36]. According to the Danish legislation on experimental animals, no permission is required to collect invertebrates (octopuses being the exception) in Danish National waters. Under laboratory conditions (16°C; 35 psu) *O* *. dioica* produces 4-6 houses d^-1^ [37] and then each house is used about 5 hours before being discarded.

In order to obtain sinking speeds of discarded houses loaded with the 4 different ballasting materials the experimental setup followed 3 steps(1). Appendicularians were first placed in a suspension of the ballasting material, thus producing discarded houses loaded with this material(2). Discarded houses were next allowed to age and deflate, and (3) their sinking speeds were finally measured. The same 0.2 µm-filtered seawater was used over the course of the experiment ensuring similar salinity and temperatures conditions.

For each type of ballasted particle we used three different concentrations of ballast particles, representative of a monospecific bloom or an intense Saharan dust event, to load the discarded houses: a medium concentration, representing meso- to eutrophic conditions (20 000 cells ml^-1^ for *I* *. galbana* and *T. pseudonana*), a lower (0.5 × medium), and a higher concentration (1.5 × medium). These concentrations correspond grossly to pre-bloom, beginning of bloom, and bloom conditions as recorded in coastal temperate environments [38]. The concentrations of Saharan dusts and *E* *. huxleyi* were obtained based on equivalent particles volume concentrations. Medium concentration of *E* *. huxleyi* (18 000 cells ml^-1^) corresponds to bloom condition [15] while medium Saharan dusts concentrations corresponded to 5-12 mg L^-1^, which is equivalent to a strong Saharan dust rain event [39]. Particle concentrations were measured using a coulter counter both at the beginning and the end of incubations, and mean concentration over the experiments were reported (Table 1).

**Table 1:** Type, initial, final and mean concentration of ballast particles used to produce appendicularian houses and recorded sinking properties of those houses.

Type of particles and concentration used		Particulate volume concentration (µm^3^ ml^-1^)			n	Mean house size		Mean sinking speed		Mean excess density	
		initial	final	mean		(mm ± std)		(m d^-1^ ± std)		(mg cm^-3^ ± std)	
Organic	0.5	8.52 10^5^	6.67 10^5^	7.60 10^5^	10	1.80	± 0.31	45.29	± 25.53	0.36	± 0.20
	1	1.27 10^6^	6.81 10^5^	9.76 10^5^	10	1.89	± 0.49	89.98	± 28.66	0.78	± 0.33
	1.5	1.61 10^6^	1.38 10^6^	1.50 10^6^	10	2.61	± 0.46	75.90	± 34.09	0.31	± 0.10
Opal	0.5	1.15 10^6^	7.64 10^5^	9.57 10^5^	10	2.12	± 0.38	82.78	± 39.65	0.39	± 0.26
	1	1.44 10^6^	1.01 10^6^	1.23 10^6^	10	2.35	± 0.44	110.94	± 39.61	0.59	± 0.14
	1.5	1.87 10^6^	1.44 10^6^	1.66 10^6^	10	2.37	± 0.37	125.20	± 30.44	0.68	± 0.13
Calcite	0.5	6.47 10^5^	5.21 10^5^	5.84 10^5^	10	1.97	± 0.48	108.05	± 38.98	0.88	± 0.37
	1	1.15 10^6^	8.51 10^5^	1.00 10^6^	10	1.98	± 0.43	141.22	± 47.95	1.02	± 0.59
	1.5	1.63 10^6^	1.32 10^6^	1.47 10^6^	10	2.13	± 0.58	167.39	± 62.10	1.23	± 0.42
Lithogenic	0.5	8.99 10^5^	2.26 10^5^	5.63 10^5^	10	1.77	± 0.51	139.12	± 75.82	1.40	± 0.80
	1	1.64 10^6^	8.93 10^5^	1.27 10^6^	10	2.70	± 0.66	159.46	± 51.03	0.79	± 0.30
	1.5	1.94 10^6^	7.53 10^5^	1.35 10^6^	10	1.79	± 0.48	230.72	± 53.27	2.58	± 0.85
Kruskall-Wallis test						X_9,90_ = 18.86		X_3,90_ = 40.91		X_3,90_ = 46.74	
						*p* = 0.026		*p* < 0.001		*p* < 0.001	

Kruskall-wallis test between the different conditions examined is also indicated.

For each condition, 40 5-day old appendicularians were picked from the mother culture and placed in a 5-L bucket filled with 0.2-µm filtered seawater to which was added the ballast particles at the targeted concentration. The buckets were then manually strongly stirred to force the appendicularians to discard their house and secrete a new one. Subsequently the buckets with appendicularians were stirred gently and continuously using internal paddles (8 rpm) for 5 hours, which is sufficient time for the animals to have houses nearly ready to be discarded [37]. Ten appendicularians with houses loaded with particles and ready to be discarded were then placed in petri dishes filled with the same water as used for the incubation until they discarded their houses. Visual observation with dissecting microscope confirmed that there were no aggregation of any ballast particles in the bottom of the bucket, suggesting that all were affected similarly by the different stirring.

The freshly discarded houses were transferred using a wide mouth pipette to a 250-mL closed bottle placed on a rotating table with a rotation speed assuring minimal contacts of the house with the bottles walls. These incubations were done using 0.2-µm filtered seawater, preventing any additional fixation of particles through scavenging [26]. The houses were left for 2 hours, which is a sufficient time to pass the strong initial deflation process [34], thus allowing comparable measurement of sinking speed between houses. Finally, houses were recovered from the bottle using a wide bore pipette and placed in a Plexiglas chamber (dimensions 5x5x20 cm) illuminated with a red laser sheet. Here their sinking speed and size were recorded using video observations, and their excess density was calculated using the Newton-Rittinger equation as described in [34].

## Results

The median size of appendicularian houses (2.11 mm, Table 1) was not strongly significantly different between experimental conditions (Kruskall-Wallis test). Therefore, the sinking speed measurements obtained between conditions can be directly compared, even if the house size variability may have increased the overall variability of sinking speed measurements (Table 1)

**Figure 1:**
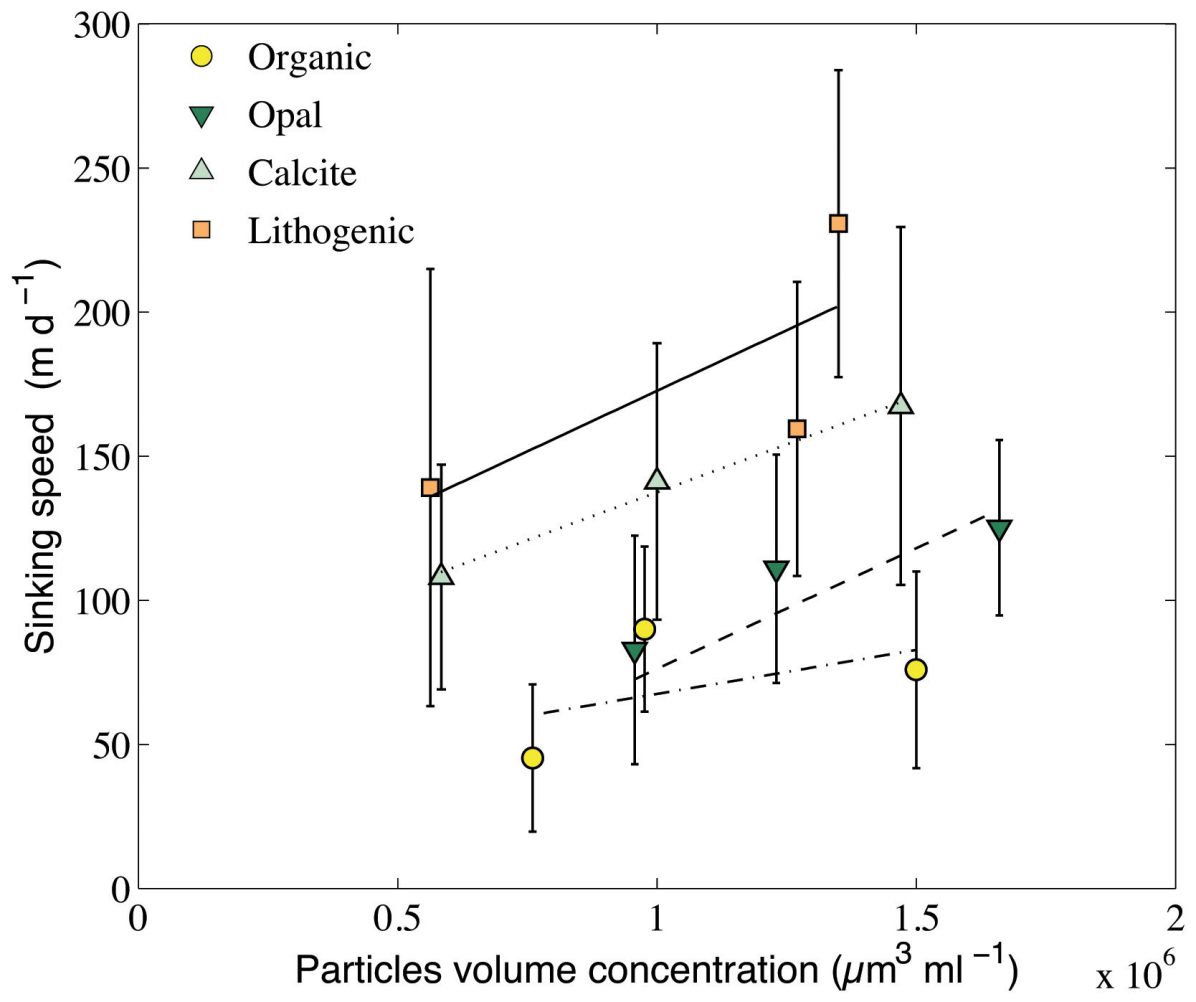
Effect of concentration and type of ballast particles on appendicularian houses sinking speed. Concentrations of ballast particles correspond to concentration in the seawater in which the appendicularians were incubated and houses produced. Mean and standard deviations for each observation (n=10). Lines and dashed lines do represent the relationships between particles volume concentration (V) and sinking speed (S) with S = 3.04 ×10^-5^ V + 37.17 for organic particles; S = 8.38 ×10^-5^ V -7.82 for opal particles; S = 6.67 ×10^-5^ V + 70.82 for calcite particles; and S = 8.38 ×10^-5^ V + 88.63 for calcite particles. The slopes of all relationships were not significantly different (mean slope of 6.65×10^-5^ m d^-1^ µm^-3^ ml; covariance analysis, F_3,111_ = 0.75, *p* = 0.52), to the contrary of the intercepts (covariance analysis, F_3,114_ = 28.9, *p* < 0.001) to the exception of organic and opal ballasted particles (covariance analysis, F_1,58_ = 3.16, *p* = 0.08).

Sinking speeds differed significantly between the differently ballasted particles (Kruskall-Wallis test; Table 1) even if the sinking speed recorded between organic ( *I* *. galbana* ) and opal particles (*T. pseudonana*) were not significantly different (pairwise comparison test). Houses loaded with organic and opal material settled with a mean sinking speed of 88 m d^-1^, while houses loaded with calcite ( *E* *. huxleyi* ) and lithogenic particles sank, respectively, at average speeds of 138 and 176 m d^-1^. Because particles concentrations changed during the course of the experiments due to appendicularian feeding activity (Table 1), mean concentration may better represents the overall concentration during the experiment; however, even using initial or final concentration do not change qualitatively our results. The aggregates’ load concentration had an effect on the sinking speed with higher concentration of ballasted particles in the seawater promoting a higher sinking speed (Figure 1; all relationships except for organic particles are significantly different from zero) with a nearly constant effect of 33 m d^-1^ settling speed increase for a 5×10^5^ µm^3^ ml^-1^ increase in particle concentration. Generally, calcite increased the sinking speeds of organic particles by ~100% and lithogenic material increases it by ~150%.

Similarly, the excess density calculated from the size of the appendicularian houses and their sinking speed (Table 1) was significantly different between the differently ballasted aggregates with the exception of organic compared to opal and calcite compared to lithogenic particles (pairwise comparison tests). Overall, organic particles and particles ballasted with opal were characterized by an excess density of 0.52 while calcite and lithogenic particles had an excess density of 1.32.

## Discussion

This is the first study to test simultaneously the impact of different ballasting material and their concentrations on the sinking speed of biogenic aggregates such as appendicularian houses. To our knowledge, only few experiment have been performed in a similar set-up to examine the impact of different minerals concentrations on aggregates size, weight, density and composition [40-42], and the effect on sinking speed was only tested in one case [41]. We first show that the concentration of ballasting material in seawater has a significant effect on the sinking velocity of aggregates produced by appendicularians. Appendicularians only ingest a minor proportion of the filtered particles [43] and discard the non-ingested particles together with the clogged houses [23]. This mechanism leads to a load of ballast into the house that is proportional to the particle concentration in the surrounding seawater [26], thereby correlating ballast concentration and house sinking speed.

The present study provides new observations on the impact of four different ballasting materials on sinking speed of marine aggregates produced by appendicularians. This is of primary importance since sinking speed is one of the major parameter controlling carbon sequestration in the ocean. Our experiment relies on the use of laboratory-made appendicularians houses, which can be a major source of marine snow in the ocean [27]. Laboratory-made aggregates made this way are likely to produce aggregates comparable to those *in situ* [34] leading to realistic estimates of sinking speeds and excess densities. Our estimates were mostly based on monospecific diets while, in the field, appendicularians may often feed on a more diverse community including organic, opal, calcite and lithogenic based particles. However, our observation may replicate monospecific blooms or intense Saharan dust rain event where one type of particles is dominating the assemblage. Our estimates are similar to those obtained for aggregates formed from natural seawater or algal cultures [9,17,44], and from *in situ* observed aggregates [7,18](Table 2). However, both our sinking speed and excess density estimates are lower than some observations using *E* *. huxleyi* cultures as the only source of particles formation [15,16,42] or different minerals in combination with diatom cultures [41] for which both sinking velocities or excess densities seem high (Table 2). This suggests that some aggregates produced artificially in laboratory from cultures of algae may be different from natural aggregates. The exact reason remains unclear since the methodology (using “old” algae cultures in rotating tanks) is similar between those two groups of studies and therefore should get the same faster remineralization of organic fraction compared to inorganic [45]. This discrepancy highlights the need to develop and compare commonly accepted sinking speed experiment methods in order to accurately constrain this key parameter on the biogeochemical models.

**Table 2:** Comparison of size, sinking velocities and excess densities observed with the inclusion of different type of ballast material incorporated with previous studies.

Ballast material incorporated	Particle size range (mm)	Sinking speed range (m d^-1^)	Excess density range (mg cm^-3^)	Reference
Organic	1-3.4	13-160	0.1-1.3	This study
Opal	1.2-3	23-191	0.14-0.92	
Calcite	1-2.9	45-283	0.3-2.3	
Lithogenic	1-3.7	41-322	0.26-4.44	
Natural aggregates*	0.3-0.6	160-280	ns	[21]
Natural aggregates*	1-20	20-200	0.01-10	[7]
Calcite+saharian dusts (formed from natural seawater)	1-6	100-600	ns	[9]
Opal	1-3	55-350	0.63-2.23	[17]
Calcite	1-2	25-63	3.36	
Appendicularian fecal pellets*	0.5-0.7	500-900	180-320	
Copepod fecal peletts	0.1	100-200	110-190	
Opal	1-5	40-200	ns	[16]
Calcite	1-4	100-325	ns	
Opal+calcite	1-4	50-200	ns	
Calcite (several concentrations)	0.9-4.5	ns	0.2-56	[45] in [34]
Calcite - low CO_2_	2-4	850-1700	0.008-8	[19]
Calcite - medium CO_2_	2-3	432-1000	0.008-9	
Calcite - high CO_2_	2.5-5	170-600	0.008-10	
*E* *. huxleyi*				[20]
Calcite	0.7-2.4	86-1800	2.1-41	
Organic	0.8-11	950-2160	0.02-15	
Opal+lithogenic	0.08-0.6	200-400	2-100	[44]
Opal+lithogenic	0.1-0.8	300-800	2-200	
Opal+lithogenic (various origin)	0.1-0.6	200-1000	1-100	

* in situ formed aggregates

The ballast type affects the sinking speed of appendicularian houses. Opal has a small effect on particles sinking speed, increasing it by ~50% while calcite and lithogenic (Saharan dust) material significantly increase the particles’ sinking speed by ~100 and ~150%, respectively. These differences are related to the specific ballast density, 2.71 g cm^-3^ for calcite, 2.65 g cm^-3^ for quartz, and 2.1 g cm^-3^ for opal, although the silicifying and calcifying species used here may have lower densities because they include organic matter from the phytoplankton [12]. Additionally, the aggregates studied here are a combination of house material and particles trapped inside it, which have an organic and inorganic fraction. This explains the lower than expected aggregate densities observed here.

It has been suggested that calcite has a higher carrying capacity than opal, allowing more carbon to be carried to depth on calcite particles relative to opal [12]. Therefore, the larger increase of sinking speed caused by calcite ballast may explain the more efficient transfer of organic matter to the seafloor by calcite than opal. However, this has to be taken with caution, since the higher carrying capacity for calcite may simply reflects a stronger global correlation between calcite and organic carbon and as such is a statistical description rather than an explicit mechanism [46]. Recent sediment trap data suggest that other factors, such as remineralization rates and ecosystem function, are also key in controlling the organic carbon reaching the seafloor [47,48]. Interestingly, it has been suggested that high latitude diatom-dominated areas are characterized by low transfer efficiency in the water column [47-49], which have been attributed to poorly packaged aggregates that disintegrate easily or are more easily remineralized. Alternatively, it could also represent a faster utilization of sinking material by the food chain [50], notably by zooplankton that intensively feed on diatom sinking aggregates [51], while microbial processes may control the transfer efficiency in picophytoplankton dominated regions, leading to a better transfer of sinking material [50]. Our results gives one additional explanation by showing that opal have only a minor influence on aggregates sinking speed compared to calcite or lithogenic particles, which also partly explain the more efficient transfer of organic matter to the seafloor by calcite than opal. 
